# The Relationship Between Colonoscopy Practice Style, Operator Ergonomics and Procedure Quality

**DOI:** 10.7759/cureus.45135

**Published:** 2023-09-12

**Authors:** Evren Besler, Mustafa F Celayir, Emre Teke, Cebrail Akyuz, Süleyman Toker

**Affiliations:** 1 General Surgery & Surgical Endoscopy, Haydarpasa Numune Training and Research Hospital, Istanbul, TUR; 2 General Surgery & Gastrointestinal Surgery, Sisli Hamidiye Etfal Training and Research Hospital, Istanbul, TUR; 3 General Surgery & Gastrointestinal Surgery, Haydarpasa Numune Training and Research Hospital, Istanbul, TUR

**Keywords:** polyp, quality, ergonomics, colonoscopy, adenoma

## Abstract

Objective: We aimed to compare three endoscopy operators who performed colonoscopy in three different styles in terms of procedure results, colonoscopy quality, and operator comfort during the procedure.

Patients and methods: A total of 246 patients, who underwent routine screening colonoscopy for precancerous lesions between May and December 2022 in Istanbul Haydarpaşa Numune Education and Research Hospital, Istanbul, Turkey, were prospectively analyzed. The results of three different styles (*single operator sitting, single operator standing*, and *two operators standing*) were compared with each other. The following criteria were examined: polyp/adenoma detection rate, number of polyps detected per patient, cecal intubation rate, total procedure time, number of endoscope corrective maneuvers, number of patient position corrections during the procedure, and the endoscopist's subjective pain scale after the procedure.

Results: The number of corrections and changes in scope position, rates of changing the patient's position during the procedure, and the postprocedural fatigue degree of the endoscopist were the highest for the single-operator standing style (*p<0.001*). The total processing time and post-procedure fatigue degree of the endoscopist were the lowest for the single-operator sitting style (*p<0.001*). The adenoma detection rate was the highest for single-operator standing style (*37.8% vs 22.0% *and* 29.3%*). The strongest predictive factors for the total procedure time were the colonoscopy style and patient age. The strongest predictive factors for the change in the total number of detected polyps were colonoscopy style, patient gender, and patient age. Independent of all other factors, the total detected number of polyps was statistically significantly higher for the single-operator standing style compared to other styles (*B=-0.217, 95% confidence interval: -0.369 - -0.066 and p=0.005*) (*B=-0.172, 95% confidence interval: -0.326 - -0.017 and p=0.029*).

Conclusions: Two conclusions were drawn from this study. For routine screening colonoscopy, the single-operator sitting style seems to be superior to other styles in terms of the shortest procedure time and the least tiring. However, the widely used single-operator standing style should be preferred over other styles in terms of the highest adenoma detection rate although it is most tiring and time-consuming.

## Introduction

Screening colonoscopies reduces the incidence of colorectal cancer (CRC) [1]. The quality of a screening colonoscopy can differ depending on various factors, such as adequate bowel cleansing, cecal intubation rate (CIR), adenoma detection rate (ADR), adenoma miss rate (AMR), scope insertion/withdrawal time, endoscopist proficiency, endoscopist fatigue, and daily screening schedule. The ADR has been accepted as an independent quality indicator for colonoscopy [2,3]. ADR is calculated by dividing the number of colonoscopies with at least one adenoma detected by the number of all colonoscopies [4]. An adequate ADR for asymptomatic individuals ≥50 years undergoing screening colonoscopy is accepted as ≥30% in men and ≥20% in women [5]. Colonoscopy quality is still highly dependent on the operator’s experience and specialty for the detection of precancerous lesions [6]. In this prospective clinical study, we aimed to compare the ADR of three different colonoscopy applications as the primary aim. As a secondary aim, we aimed to determine the correlation between the endoscopy fatigue level and the colonoscopy application method. With this study, we aimed to determine the "optimal application style" in which the entire colon can be examined in the shortest time, with the highest ADR and with the least operator fatigue.

## Materials and methods

**Figure 1:**
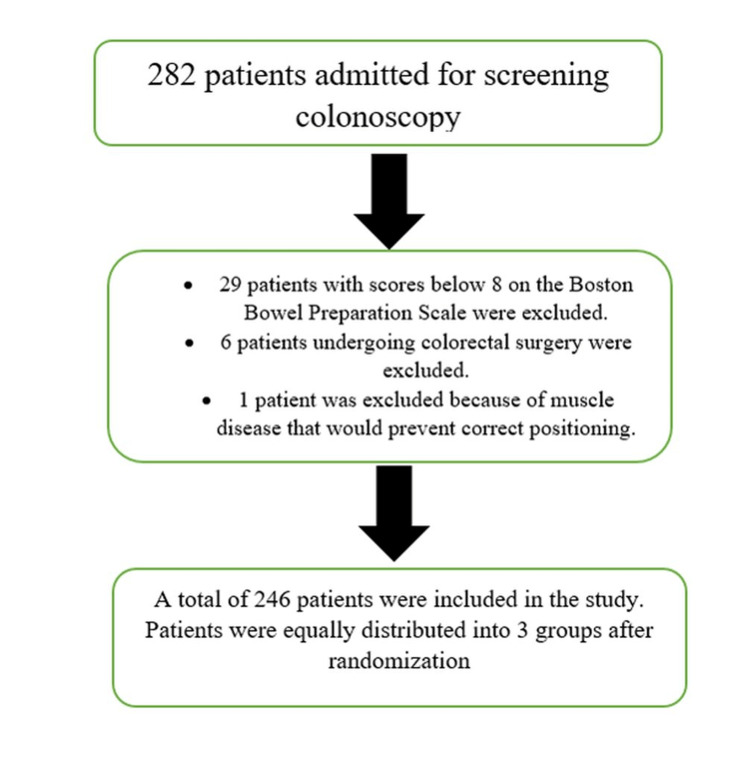
Flow diagram

Two hundred forty-six patients, who underwent screening colonoscopy for detecting polyps, precancerous lesions, and cancer at the Surgical Endoscopy Unit of Haydarpaşa Numune Education and Research Hospital/Istanbul/Turkey between May and December 2022, were prospectively evaluated. A flow diagram is shown in Figure 1.

Ethical statement: Institutional Review Board/Ethics Committee approval (Decision No: 2022/KK/81, date: 18/04/2022) and Institute Chief Physician approval (decision No: 62977267-E.79, date: 22/04/2022) were received for this study from the Ethics Committee and Hospital Management of TR Health Sciences University - Istanbul Haydarpaşa Numune Training and Research Hospital.

Patient distribution: Patients were randomized and assigned to endoscopists via the www.randomizer.org website. Routine patient screening colonoscopies to be performed by the operators with three different application styles were recorded with daily data sheets. Informed consent forms were obtained from the patients. Identifiable information about the patients who underwent the procedure was kept confidential.

Patient inclusion criteria: The inclusion criteria were as follows: all elective patients with full bowel preparation according to the Boston bowel preparation scale (BBPS) eight or above and aged 18-80 years who had a routine appointment for full screening colonoscopy under anesthesia. Only procedures performed under anesthesia were included in the study to establish a standard reaction scale regarding the effect of patients' pain response on the duration of the procedure.

Patient exclusion criteria: The exclusion criteria were as follows: patients undergoing procedures without anesthesia, patients undergoing planned partial colonoscopic examination and/or recto sigmoidoscopy, patients who had previous abdominal surgery or colorectal surgery for any reason, patients with a stoma, patients requiring colonoscopy for emergency intervention (acute gastrointestinal bleeding, etc.), and patients with any musculoskeletal disorder that may prevent position change on the colonoscopy table.

Operator characteristics: Three surgical endoscopists with different application styles, each performing ≥300 colonoscopies/year with at least five years of experience, were included in the study. It was only a researcher-blind study, as both the patient and the endoscopist saw the procedure. All three operators did not have any musculoskeletal disorders or any other chronic diseases that would affect the quality of the procedure. All three endoscopists were examined by a doctor who specialized in physical therapy and rehabilitation for musculoskeletal conditions. The application styles were as follows: 1) single operator sitting (1op-sit), 2) single operator standing (1op-sta), and 3) two operators standing (2op-sta). These application styles were classified as "Group 1, Group 2, and Group 3," respectively.

Description of the procedure: All procedures started with the left lateral decubitus position of the patient. Supine and right lateral decubitus positions were applied in some cases. Surgical endoscopy nurses attended to all procedures. In assisted procedures (2op-sta), the surgical nurse additionally kept the flexible fiber scope for performing the forward, backward, and rotational movements of the scope.

For each of the three application styles, the widely used quality of colonoscopy parameters in the literature was examined: 1) total (insertion + withdrawal) process time (TPT), 2) CIR, and 3) ADR. Withdrawal time (WT) is a standard benchmark for colonoscopy quality [7]. However, in some studies, it has been stated that the scope insertion phase is also important and contributes to ADR [8-11]. In this study, TPT was examined instead of WT with support of literature knowledge, because in some cases some of the polyps/adenomas were picked up at the insertion phase of the colonoscopy depending on the practical habit and preference of the operator. If a polyp was detected during the procedure, an additional nurse was included in the procedure.

Additional parameters indicating the ergonomics and fatigue of the operator were examined: 1) number of maneuvers to correct/reposition the endoscope, 2) number of patient position changes during the procedure, and 3) the endoscopist's total mean waist, neck, back, knee fatigue/pain scale (on the basis of subjective measurement between 1-10) after the procedure. The traditional Visual Analogue Scale (VAS) was used for practitioner fatigue. Only due diligence (yes-no) was made with other secondary titles.

Situations requiring extra attention during the procedure included perioperatively detected tumors and diverticulitis. Additional features requiring extra attention included excessive looping that made it difficult to reach the cecum, long colon anatomy, and a sagging abdominal wall. It is obvious that these extra situations would prolong the total processing time. There was no luminal iatrogenic perforation in any of the patients included in the study.

Sample size estimation

Power analysis and number of cases: In the power analysis for the ANOVA test, when the power of the test (1-β) = 0.80, the error level (α) = 0.05, and the effect size = 0.20, it was determined that 246 patients were needed for the study.

Statistical analysis

Data were analyzed with the IBM SPSS Statistics 25.0 (IBM Corporation, Armonk, NY, USA) package program. Results were considered statistically significant when p<0.05.

Other tests used: The Kolmogorov‒Smirnov test was used to determine whether the distribution of discrete and continuous numerical variables was close to normal. Levene's test was used to determine whether the assumption of homogeneity of variances was satisfied. Descriptive statistics are expressed as the mean ± standard deviation or median (minimum‒maximum) for discrete and continuous variables, while categorical variables are presented as the number of cases and proportions (%). Student’s t-test was used to compare normally distributed continuous variables across two independent groups; one-way ANOVA was used to compare normally distributed continuous variables across more than two independent groups. The Mann‒Whitney U test was used to compare nonnormally distributed continuous variables across two independent groups; the Kruskal‒Wallis test was used to compare nonnormally distributed continuous variables across more than two independent groups. According to our current findings, the primary endpoints of the study were whether it reaches the cecum, the total procedure time, and the number of polyps detected. The most important independent variable of the study is the method of colonoscopy application, and variables such as age, gender, and additional characteristics are other independent variables. If the Kruskal-Wallis test statistic results were found to be significant, the Dunn‒Bonferroni test was used to determine which groups were significantly different. Pearson's χ2 test was used to compare categorical data, unless otherwise stated. On the other hand, if the expected frequency was below five in at least ¼ of the cells in the 2x2 crosstabs, the categorical data were evaluated with Fisher's exact probability test, while the χ2 test with continuity correction was used when the expected frequency was between five and 25. To analyze the categorical data in the crosstabs of RxC (in the case at least one of the categorical variables in the row or column had more than two results), the Fisher‒Freeman‒Halton test was used if the expected frequency was below five in at least ¼ of the cells. Spearman's correlation analysis was used to evaluate whether there was a statistically significant correlation between age, the total duration of the procedure, and the total number of polyps detected. Multivariate logistic regression analysis was performed to examine the effect of the colonoscopy method on the inability to reach the cecum after adjusting for age, sex, and additional characteristics of the patient. Multivariate linear regression analysis was used to examine the effect of the colonoscopy method on predicting the change in total procedure time and the total number of polyps detected. Logarithmic transformation was applied in linear regression analyses since the data of total procedure time and total number of polyps detected were far from normal. Results were considered statistically significant when p<0.05.

## Results

**Figure 2:**
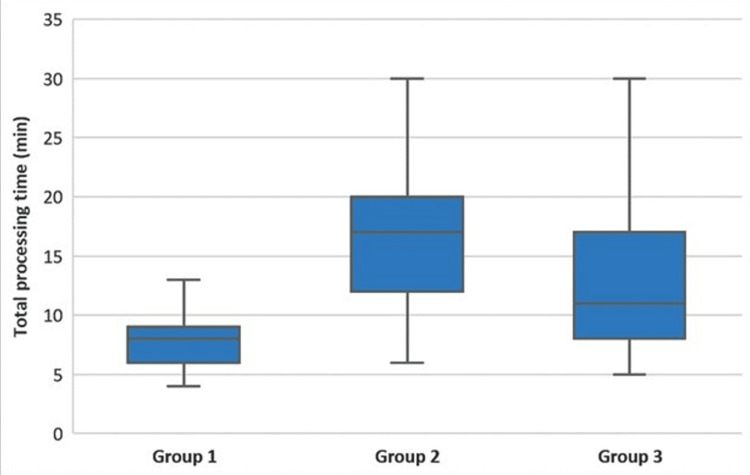
Comparison of the total processing times among the groups The horizontal lines in the middle of each box indicate the median, while the top and bottom borders of the box mark the 25th and 75th percentiles, respectively. The whiskers above and below the box mark the maximum and minimum processing times.

There was a statistically significant difference between the groups in terms of the TPT (longer in Group 2 than in Groups 1 and 3 (p<0.001), longer in Group 3 than in Group 1) (p<0.001)) (Figure 2, Table 1).

**Table 1:** Demographic and clinical characteristics of the cases according to the groups Group 1: One operator sitting (1op-sit), Group 2: One operator standing (1op-sta), Group 3: Two operators standing (2op-sta). Descriptive statistics are shown as * mean ± standard deviation or ** median (minimum-maximum). † One-way ANOVA, ‡ Pearson’s χ2 test, ¶ Kruskal‒Wallis test, ¥ Fisher‒Freeman‒Halton test. a: The difference between Groups 1 and 2 was statistically significant (p<0.001), b: The difference between Groups 1 and 3 was statistically significant (p<0.05), c: The difference between Groups 2 and 3 was statistically significant (p<0.05).

	Group 1 (n=82)	Group 2 (n=82)	Group 3 (n=82)	p-value
Age (yrs)*	54.8±13.9	56.8±12.9	57.7±13.3	0.354†
Gender				0.113‡
Male	43 (52.4%)	30 (36.6%)	39 (47.6%)	
Female	39(47.6%)	52 (63.4%)	43 (52.4%)	
Total processing time (min)**	8 (4-20)^a,b^	17 (6-30)^a,c^	11 (5-30)^b,c^	<0.001¶
Cecal intubation rate	75(91.5%)	74 (90.2%)	72 (87.8%)	0.732‡
Inability to intubate the cecum	7 (8.5%)	8 (9.8%)	10 (12.2%)	0.732‡
Adenoma detection rate	18 (22.0%)	31 (37.8%)	24 (29.3%)	0.084‡
Mean number of polyps detected per patient*	0.39±0.96	0.85±1.63	0.52±1.13	0.059†
Number of maneuvers to correct/reposition the endoscope**	0 (0-4)^a,b^	4 (0-15)^a,c^	2 (0-4)^b,c^	<0.001¶
Number of patient position changes during the procedure	1 (1.2%)^a,b^	40 (48.8%)^a,c^	24 (29.3%)^b,c^	<0.001‡
Post-procedure endoscopist fatigue**	3 (2-8)^a,b^	4 (1-8)^a^	4 (1-8)^b^	<0.001¶
Situation requiring extra attention during the procedure	6 (7.3%)^b^	5 (6.1%)	0 (0.0%)^b^	0.032¥
Additional features	9 (11.0%)^b^	13 (15.9%)^c^	0 (0.0%)^b,c^	<0.001‡

There was a statistically significant difference between the groups in terms of the number of corrections and changes in the scope position (higher in Group 2 than in Groups 1 and 3 (p<0.001) and higher in Group 3 than in Group 1 (p<0.001)) (Table 1).

The frequency of changing the patient's position during the procedure was significantly higher in Group 2 than in Groups 1 and 3 (p<0.001 and p=0.010, respectively). In addition, the frequency of changing the patient's position during the procedure in Group 3 was significantly higher than that in Group 1 (p<0.001) (Table 1).

The postprocedural fatigue degree of the endoscopist was significantly higher in Groups 2 and 3 than in Group 1 (p<0.001 and p=0.012) (Table 1).

There was a statistically significant difference between the groups in terms of requiring extra attention during the procedure (p=0.032), and the reason for this difference was that the situation requiring extra attention was encountered more frequently in Group 1 than in Group 3 (p=0.028).

There was a statistically significant difference between the groups in terms of the presence of additional features (p<0.001), and the reason for this difference was the higher proportion of patients with additional features in Groups 1 and 2 than in Group 3 (p=0.003 and p<0.001, respectively) (Table 1).

**Figure 3:**
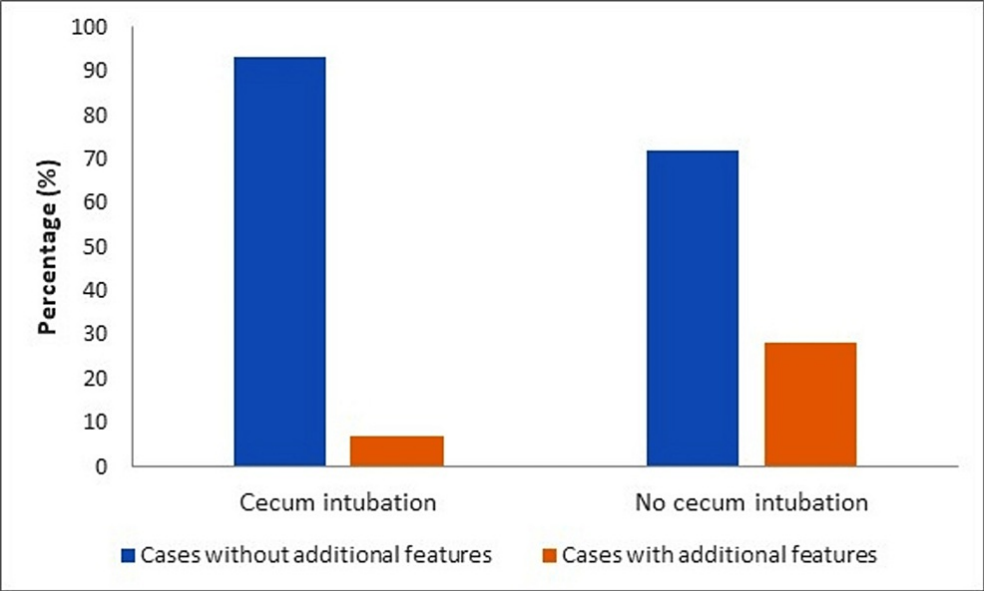
Cecal intubation rate relationship with additional features

While there was no statistically significant difference between the group in which the cecum could be reached and the group whose cecum could not be reached (p=0.539 and p>0.999), the rate of those with additional features was statistically significantly higher in the group whose cecum could not be reached than in the group whose cecum could not be reached (p=0.003) (Figure 3, Table 2).

**Table 2:** Demographic and clinical characteristics of the cases according to the groups with and without access to the cecum † Student’s t test, ‡ χ2 test with correction for continence, ¶ Fisher's exact probability test.

	Cecum intubated (n=221)	Cecum not intubated (n=25)	p-value
Age (yrs)	56.3±13.4	58.0±13.5	0.539†
Gender			>0.999‡
Male	101 (45.7%)	11 (44.0%)	
Female	120 (54.3%)	14 (56.0%)	
Additional features	15 (6.8%)	7 (28.0%)	0.003¶

After adjusting for other factors, the likelihood of inaccessibility to the cecum increased in patients with any additional feature rather than in patients without any additional feature (OR=7.910, 95%CI: 2.426-25.786, p<0.001) (Table 3).

**Table 3:** Examination of the combined effects of all possible factors thought to be effective on inaccessibility to the cecum – results of multivariate logistic regression analysis Group 1: One operator sitting (1op-sit), Group 2: One operator standing (1op-sta), Group 3: Two operators standing (2op-sta). The R square value of the model was relatively low and was obtained as 9.7%.

	Odds ratio	95% Confidence interval	Wald	p-value
Age	0.999	0.967–1.031	0.007	0.935
Female sex	1.006	0.423–2.396	0.000	0.989
Additional features	7.910	2.426–25.786	11.764	<0.001
Group 1	1.002	0.321–3.125	0.000	0.997
Group 3	2.338	0.751–7.279	2.149	0.143

While there was no statistically significant difference between men and women in terms of TPT (p=0.449), the total number of polyps detected in men was statistically significantly higher than that in women (p=0.042) (Table 4). TPT was significantly longer in the group with additional features than in the group without additional features (p=0.023) (Table 4). As age increased, the TPT increased significantly (r=0.211 and p<0.001). In addition, there was a statistically significant and same-sided association between age and the total number of polyps detected (r=0.181 and p=0.004) (Table 4).

**Table 4:** Total processing time and total number of polyps detected by gender and additional features Descriptive statistics; displayed in median (minimum‒maximum) format. † Mann‒Whitney U test.

	Total processing time (min)	Total number of polyps detected
Gender		
Male	11 (4-30)	0 (0-10)
Female	11 (4-30)	0 (0-4)
p-value †	0.449	0.042
Additional features		
Absent	11 (4-30)	0 (0-10)
Present	14.5 (4-25)	0 (0-4)
p-value †	0.023	0.915

The "most determinant" factors in estimating the TPT were the colonoscopy style and patient age. Independent of other factors, if the number 1 (B=-0.712, 95% confidence interval: -0.833 - -0.591 and p<0.001) and number 3 (B=-0.343, 95% confidence interval: -0.467 - -0.220 and p<0.001) styles were applied, the TPTs continued to shorten statistically according to style 2. In addition, independent of other factors, TPT was significantly longer with advanced age (B=0.006, 95% confidence interval: 0.002-0.010 and p=0.002) (Table 5).

**Table 5:** Examination of the combined effects of all possible factors that are thought to be determinative in estimating the total procedure time and the change in the total number of detected polyps – results of multivariate linear regression analysis Group 1: One operator sitting (1op-sit), Group 2: One operator standing (1op-sta), Group 3: Two operators standing (2op-sta). The R square value of the model for the total processing time was 8.1%, and the R square value of the number of polyps was 8.3%.

	Regression coefficient	95% Confidence interval	t-statistics	p-value
Total processing time				
Age	0.006	0.002–0.010	3.204	0.002
Female sex	-0.031	-0.130–0.069	-0.610	0.543
Additional features	0.099	-0.080–0.278	1.094	0.275
Group 1	-0.712	-0.833 – -0.591	-11.572	<0.001
Group 3	-0.343	-0.467 – -0.220	-5.472	<0.001
Total number of polyps detected				
Age	0.007	0.002–0.012	2.972	0.003
Female sex	-0.204	-0.328 – -0.080	-3.247	<0.001
Additional features	-0.083	-0.306–0.141	-0.729	0.467
Group 1	-0.217	-0.369 – -0.066	-2.830	0.005
Group 3	-0.172	-0.326 – -0.017	-2.193	0.029

The "most determinant" factors in estimating the change in the total number of detected polyps were sex, age, and colonoscopy style used. Regardless of other factors, fewer polyps were detected in women than in men (B=-0.204, 95% confidence interval: -0.328 - -0.080 and p<0.001). In addition, when adjusted for other factors, the total number of polyps detected increased statistically with advanced age (B=0.007, 95% confidence interval: 0.002-0.012, and p=0.003).

Finally, independent of other factors if the 1op-sit (B=-0.217, 95% confidence interval: -0.369 - -0.066 and p=0.005) and 2op-sta (B=-0.172, 95% confidence interval: -0.326 - -0.017 and p=0.029) styles were applied, the total number of polyps was statistically significantly lower according to 1op-sta style (Table 5).

## Discussion

A safe, comfortable, and effective colonoscopy is not possible without natural talent and practical skill [12]. Since each patient will have different needs and each colon will be a unique challenge, the procedure requires patience and experience [13]. In 2014, Lee et al. described the three-application style of colonoscopy [14]. According to this, 1op-sta is mostly preferred and the most tiring style compared to the other two styles. 1op-sit is the least preferred, least tiring style compared to the other two styles. 2op-sta is low tiring and has a relatively high risk of perforation. If the goal of screening colonoscopy is to detect as many precancerous lesions as possible, all physical maneuvers to fully examine the entire colon should be discussed.

Intubating the colon with less insufflation and shortening it by removing loops would result in faster cecal intubation. The 1op-sit style cecal insertion technique is just described because it allows less operator body movement range due to the sitting position. This style is a more detailed and technical style that includes specific minor hand maneuvers (preferably using torque steering rather than wheel steering, "scissor-elbow-thigh (SET)” maneuvers, etc.) that provide the least operator fatigue [15]. In addition, colonoscopy performed by the operator in the sitting position was defined as effective and safe as the standing position, and it was stated that it provides additional sitting comfort [16]. This technique may have been preferred by the operator over the years of experience due to its comfort, shorter process time, and being less tiring. Although this style seems preferable with its current features, it becomes controversial due to our low ADR results. The lowest ADR of the 1op-sit style may be associated with the shortest mean TPT and relatively shortest mean cecal insertion time.

The 1op-sta and 2op-sta styles allow the operator and/or assistant more body movement range, thus resulting in more free movement range of the scope.

1op-sta is the most preferred style among endoscopists. This may be due to the short learning curve and the way endoscopy educators generally perform the procedure with this style. This style is unsurprisingly the most tiring one because all maneuvers and the whole scope are under the control of a single-standing operator. Compared to 1op-sit, this style may have more rough scope movements that trigger more endo-luminal looping and relatively higher perforation risk. When compared to the other two styles, the relatively high number of maneuvers to correct/reposition the endoscope, the number of patient position changes during the procedure, and post-procedure endoscopist fatigue support this argument. Various studies have shown that changing the patient's position during the procedure contributes positively to ADR [17-19]. Undoubtedly, all these maneuvers would lead to extra operator fatigue. Moreover, chronic fatigue will undoubtedly reveal musculoskeletal disorders over time. In a survey of colorectal colonoscopist physicians, six of 226 operators required surgery for colonoscopy-related injuries, including cervical disc and carpal tunnel injuries [20]. Because of its particular prevalence among endoscopists, the “colonoscopist's thumb” has been described [21]. Park has remarked on the importance of proper technique and maintenance of proper body posture during the procedure to reduce the risk of endoscopy-related injuries [22]. However, there are some other studies indicating that endoscopist fatigue does not affect the ADR [23]. In a retrospective study including 34,022 cases, it was concluded that endoscopists’ daily fatigue negatively affected colonoscopy quality [24]. The results of our study in the 1op-sta group with the highest ADR and high CIR rates (37.8% and 90.24%, respectively) support these last two conclusions.

The 2op-sta style may be comfortable for some endoscopists because of controlling only the knob of the colonoscope with two hands and participation of lesser body movements. Therefore, this style is expected to be less tiring for the endoscopist. However, the lack of physical resistance and tactile sensation of the scope brings the risk of perforation. This style is preferred by a minority of endoscopists [25]. Some studies report that nurse-assisted endoscopy is more comfortable and does not prolong the duration of the procedure [26]. Quality results of this processing technique have been reported in publications more or less similar to 1op-sta [25,27]. However, our results (2op-sta group with 29.26% ADR and 87.80% CIR compared to the 1op-sta group with 37.8% ADR and 90.24% CIR, respectively) differed slightly from this argument.

Lund et al. examined the quality parameters of colonoscopy in a systematic retrospective review study, including 616,390 screening colonoscopies performed by 1,431 colonoscopists and 2,319 subsequent interval CRCs. It was concluded that an optimal individual colonoscopist with a mean WT of >6 minutes, a CIR of 90%, and an ADR of 15-19% or better ≥25% should be preferred to minimize the risk of interval CRC [28]. Zhao et al. stated that 9 minutes of WT was recommended for both high ADR and low AMR without reducing the efficiency of screening colonoscopy [29]. Taking this argument one step further, Kashiwagi et al. recommend a WT "longer than" 9 minutes to examine the transverse and sigmoid colon due to the anatomical features of mobility of these segments and the possibility of missing polyps [30]. The contribution of the long examination time to the quality of colonoscopy seems indisputable. However, the daily schedule and the time pressure of the work team on the operator would make the operator go faster out of necessity. In particularly difficult cases, optimal scanning of the entire colon is up to the skill and conscience of the operator. When the goal is a high-quality colonoscopy and high ADR, is the rest not important? To provide an optimal answer to this question, multiple factors such as the number of daily procedure appointments, endoscopist experience, rate of unusual cases, and rate of interval CRC should be evaluated together.

Limitations of our study

The sampled polyps were not classified according to their sizes. Patients with a sagging abdominal wall were not evaluated as a fatigue parameter. Interval CRC results could have been analyzed for a full-quality parameter. A more effective operating style evaluation could have been performed with these and broader parameters. Not evaluating the level of patient satisfaction was another limitation of our study.

## Conclusions

Colonoscopy practice requires patience, experience, skill, and optimization of the processing time. With detailed statistical analyses, two conclusions were drawn from this study. The single-operator sitting style is superior to other styles for routine screening colonoscopy because it has the shortest overall procedure time and is more technical - least tiring. The most widely used style of single operator standing is the most tiring and most time-consuming one, but it should be preferred for its highest polyp detection rate compared to other styles. Further studies with more parameters and larger series on the ergonomics of endoscopists will contribute to the subject.

## Appendices

Recent presentation: This study was presented as an oral paper (No: S-096) in II. International Colorectal Surgery Congress, XIX. National Colon and Rectal Surgery Congress on May 16-20, 2023, at Susesi Convention Center in Antalya, Turkey.
